# Delivering a primary care-based social prescribing initiative: a qualitative study of the benefits and challenges

**DOI:** 10.3399/bjgp18X696617

**Published:** 2018-05-22

**Authors:** Kathryn Skivington, Mathew Smith, Nai Rui Chng, Mhairi Mackenzie, Sally Wyke, Stewart W Mercer

**Affiliations:** MRC/CSO Social & Public Health Sciences Unit, Institute of Health and Wellbeing, University of Glasgow, Glasgow.; MRC/CSO Social & Public Health Sciences Unit, Institute of Health and Wellbeing, University of Glasgow, Glasgow.; School of Social and Political Sciences, Institute of Health and Wellbeing, University of Glasgow, Glasgow.; School of Social and Political Sciences, Institute of Health and Wellbeing, University of Glasgow, Glasgow.; School of Social and Political Sciences, Institute of Health and Wellbeing, University of Glasgow, Glasgow.; Glasgow Caledonian University, Glasgow.

**Keywords:** community organisations, link workers, primary health care, qualitative research, social prescribing, socioeconomic factors

## Abstract

**Background:**

Social prescribing is a collaborative approach to improve inter-sectoral working between primary health care and community organisations. The Links Worker Programme (LWP) is a social prescribing initiative in areas of high deprivation in Glasgow, Scotland, that is designed to mitigate the negative impacts of the social determinants of health.

**Aim:**

To investigate issues relevant to implementing a social prescribing programme to improve inter-sectoral working to achieve public health goals.

**Design and setting:**

Qualitative interview study with community organisation representatives and community links practitioners (CLPs) in LWP areas.

**Method:**

Audiorecordings of semi-structured interviews with 30 community organisation representatives and six CLPs were transcribed verbatim and analysed thematically.

**Results:**

Participants identified some benefits of collaborative working, particularly the CLPs’ ability to act as a case manager for patients, and their position in GP practices, which operated as a bridge between organisations. However, benefits were seen to flow from new relationships between individuals in community organisations and CLPs, rather than more generally with the practice as a whole. Challenges to the LWP were related to capacity and funding for community organisations in the context of austerity. The capacity of CLPs was also an issue given that their role involved time-consuming, intensive case management.

**Conclusion:**

Although the LWP appears to be a fruitful approach to collaborative case management, integration initiatives such as social prescribing cannot be seen as ‘magic bullets’. In the context of economic austerity, such approaches may not achieve their potential unless funding is available for community organisations to continue to provide services and make and maintain their links with primary care.

## INTRODUCTION

Supporting people whose health problems are exacerbated or created by complex socioeconomic factors is a challenge to healthcare systems, especially for GPs and other primary care staff.^1^ Patients in these contexts can also be dissatisfied with the support they receive.^2^^–^6 However, GP practices in the UK are in a unique position, providing universal coverage free at the point of care in the community as part of the NHS, allowing continuity and trusting relationships to be fostered. They, therefore, provide an ideal setting to implement interventions with the potential to mitigate the impact of the social determinants of health,^7^ something that is recognised as necessary for primary health care.^8^^,^^9^ Greater collaboration between the healthcare sector and community-based provision of health and social care, with involvement of community, voluntary, and third-sector organisations (hereafter referred to as community organisations), has been promoted to help mitigate the effects of the social determinants of health.^10^^,^^11^ One such model is social prescribing, which encompasses a range of approaches.^12^ The most basic form is signposting patients to organisations without formal referral links.^13^ More comprehensive forms include referral from primary care to specific programmes such as exercise;^14^ to co-located services such as financial advice;^15^ and the use of a facilitator to develop links between patients and community organisations.^16^^,^^17^ These forms of social prescribing provide the potential for primary care teams to respond more effectively to the social determinants of health and to widen the support network accessible to people presenting at their GP practice.^18^ This approach could also potentially ameliorate some of the effects of the inverse care law operating in deprived areas.^6^^,^^19^^,^^20^

The Scottish Government-funded Links Worker Programme (LWP) is an example of a social prescribing initiative. This programme was implemented in 2014 in some of the most socioeconomically deprived areas in Glasgow.^21^ Community links practitioners (CLPs) have a third-sector or community development background and were employed to work in GP practices, taking referrals from primary care professionals, to support patients to access community organisations, with the aim of improving their health and wellbeing, and mitigating the negative impacts of the social determinants of health. A further role of the CLP is to encourage organisational change by supporting their practices to strengthen links with community organisations, therefore aiming to improve inter-sectoral working.

**How this fits in**

Supporting people whose health problems are exacerbated or created by complex socioeconomic factors is a challenge to healthcare systems, especially for GPs and other primary care staff. The Links Worker Programme, a social prescribing initiative located in general practices in the most socioeconomically deprived areas of Glasgow, was introduced to improve patient health and wellbeing, and mitigate the negative impacts of the social determinants of health, partly by improving inter-sectoral working with community organisations. This qualitative study explores the Links Worker Programme from the perspective of local community organisations. Much of the focus of existing research has been on primary care, but the involvement of community organisations, despite being a key part of social prescribing, is discussed less; this qualitative study explores the Links Worker Programme from this perspective. Although there were positive views about the programme, it is clear that fundamental issues related to the context of austerity and funding opportunities for community organisations limit the potential for sustained success.

The growing appeal of social prescribing, including at policy level,^22^^,^^23^ is juxtaposed with a lack of evidence of its effectiveness.^16^^,^^24^^,^^25^ Although much of the focus of research has been on primary care, the involvement of community organisations, despite being a key part of social prescribing, is discussed less in the literature. The success of the LWP relies somewhat on the collective action of various organisations, without which the intervention is unlikely to be implemented successfully or embedded into practice.^26^

The aim of this study was to investigate the benefits of and challenges to implementing a social prescribing programme to improve inter-sectoral working to mitigate the negative effects of the social determinants of health. Forthcoming articles (to be submitted) will report on the process and outcome evaluation of the LWP, providing broader discussion of its implementation in primary care.

## METHOD

### Study design

Qualitative methods were chosen to explore the views of CLPs and representatives of community organisations that had a connection with the LWP. Semi-structured interviews were used to facilitate exploration of predefined topics emerging from the literature, as well as issues that arose throughout the data collection and analysis.

### Sampling and recruitment

The initial phase of research involved a series of in-depth interviews with all six CLPs in the LWP, to elicit the types of challenges experienced in generating and sustaining links with community organisations. This informed subsequent interview content with community organisation participants.

The most efficient way to identify community organisations with involvement in the LWP was to do so via the CLP. Each CLP was asked to identify organisations that they had attempted to establish links with, whether successfully or where they had experienced a challenge to collaboration.

To reflect the various issues and priorities that patients identified in their consultations with CLPs (data collected in the wider evaluation of the LWP), a range of organisations were recruited in terms of their focus (Box 1). Organisations were also purposively recruited to gain a diversity of size and structure (for example, small local charities and larger charities, including those contracted to carry out services previously delivered through public sector organisations). To explore the range of issues relevant to creating links between primary care and community organisations, participants were recruited from frontline and managerial staff. The sample framework therefore reflected organisational focus, organisation type, and staff role. Based on the information from CLPs and on the sample framework, 38 community organisation representatives were approached to take part. It was intended to collect data until new themes were no longer being added to the analysis framework.

Box 1. **Details of community organisation representatives**

**Table:** 

**Number**	**Organisation type**	**Organisation focus**	**Participant job role**
1	Charity (local)	Community integration	Frontline/management
2	Statutory	Spiritual service, such as community chaplain	Frontline
3	Charity (local)	Community engagement	Management
4	Charity (national)	Cancer	Management
5	Charity (city wide)	Alcohol abuse	Frontline
6	Statutory	Advice	Frontline
7	Statutory	Social work	Frontline
8	Charity (local)	Women’s centre	Frontline/management
9	Charity (city wide)	Arts/wellbeing	Management
10	Charity (city wide)	Bereavement	Frontline/management
11	Charity (local)	Wellbeing	Frontline
12	Statutory	Psychotherapy	Frontline
13	Statutory	Employment	Frontline
14	Charity (city wide)	Alcohol abuse	Management
15	Statutory	Mental health	Frontline
16	Charity (local)	Financial advice	Frontline
17	Charity (city wide)	Dementia	Frontline
18	Statutory	Advocacy	Frontline
19	Charity (city wide)	Sexual abuse	Frontline
20	Charity (local)	Media and arts	Management
21	Charity (city wide)	Wellbeing	Management
22	Statutory	Health improvement	Frontline
23	Charity (city wide)	Wellbeing, arts	Management
24	Charity (local)	Community development	Frontline
25	Charity (city wide)	Employment	Management
26	Charity (local)	Wellbeing	Frontline
27	Charity (local)	Wellbeing	Management/frontline
28	Charity (local)	Foodbank	Management/frontline
29	Charity (city wide)	Mental health	Management/frontline
30	Statutory	Mental health	Management

### Data collection

Individual interviews, lasting approximately 60 minutes, were conducted by one of the researchers in participants’ workplaces in early 2016. All six CLPs and 30 community organisation participants were interviewed. Data saturation was reached from the perspective of the main fieldworker towards the end of data collection; no new major themes were arising, and novel data were a facet of the individual nature of the organisations rather than an aspect of themes that had not been broached. The topic guide for CLPs focused on developing links with community organisations, in particular attempting to elicit the challenges to fostering such links. The topic guide for community organisation participants was developed based on a literature review and the CLP interviews. The topics covered were: participants’ views on what the LWP can achieve and its sustainability; relationships between primary care and community services; relationships with CLPs; the referral process and appropriateness of referrals; and organisational capacity. Informed written consent was obtained from all participants. All interviews were audiorecorded and transcribed verbatim.

### Data analysis

Following the interviews with CLPs, a brief overview was developed on the main emergent themes, and these were used in the formulation of the community organisation topic guide. The main analysis was thematic, guided by framework,^27^ and included data from all interviews with CLPs and community organisation participants. Three members of the research team independently developed initial coding frameworks based on two different CLP transcripts and two different community organisation transcripts each. One coding framework was agreed through discussion and all interview data were coded in NVivo (version 10) accordingly. One researcher coded the remainder of the transcripts, and another checked the coding. To explore cases and themes, code summaries were written and patterns within each code were explored, as well as patterns across participant groups. From these patterns, central strengths and challenges of the LWP were explored.

### Participants

The results are based on the analysis of data from individual interviews with all six CLPs and 30 representatives of community organisations (Box 1). The CLPs had varied backgrounds, but with employment history in community organisations or community development. Quotes provided in the Results section are attributed to community organisation representatives as CO with the relevant organisation’s number, and to CLPs with a number relating to their practice.

## RESULTS

Participants reported benefits to collaborative working, especially more effective case management and the facilitation of community organisation presence in GP practices. Challenges in implementing and sustaining the LWP were apparent, with the context of austerity, the capacity of CLPs, and the building of individual rather than organisational relationships. It was not possible to ascertain, from those organisations that did not engage, whether there were particular barriers to connecting with the LWP in the first place, or if there were reasons why they perhaps chose not to engage. The CLPs identified some challenges to engaging with these organisations, such as not being able to get in contact with an individual to take forward the relationship. Likewise, when attempting to recruit these organisations (three in total that were identified by the CLPs) it was not possible to gain contact to arrange participation. This was because of difficulty of gaining initial contact rather than gaining interest in participation in the study. There were no clear differences in views between participants in terms of their organisations’ focus or their job role.

### Benefits of collaborative working

#### Case management

The main benefit of the LWP, from community organisation representatives’ experiences of working with patients engaged with the programme, was that it provided patients with a central point of contact. They believed that CLPs acted as a bridge between stakeholders because they have an understanding of the patient, primary care, and other available resources.

This was especially useful for engaging those who would otherwise not have benefited from services outside formal primary health care:

‘Reaching the people, I think, who are the hardest to reach. The people that don’t realise that — although they might be aware of us, they don’t realise that we could actually help them.’

(CO6)

Such patients were now finding routes to appropriate support via the CLPs’ case management role because they were receiving more dedicated time and gaining information tailored to their needs.

Most participants viewed the role of the CLP as engaging patients with a network of community resources and providing continued follow-up and support, rather than simply being a referral point. Fulfilment of this role was, unsurprisingly, thought to be contingent on local knowledge:

‘Her knowledge of all things knocks me over sometimes, you know, she has a vast knowledge of how she can help families.’

(CO3)

The benefit of the CLP, from the community organisation perspective, goes beyond supporting patients to access and engage with appropriate support — they often continue to work collaboratively with each organisation on individual patient cases:

‘There is a capacity for someone in that role, I think, to coordinate the different aspects of someone’s care, but also to have that kind of broad-based knowledge of what might be most useful for them.’

(CO12)

It was clear from many of the community organisation representatives that they thought partnerships were forming with their CLP, with clear two-way dialogue. Even in cases where participants, or their organisations, did not yet have such close collaboration with CLPs, they could appreciate the role of the programme in engaging patients with systems of support.

#### Facilitation of community organisation presence in GP practices

Community organisation participants suggested that, before the LWP, as a *‘… third sector representative or professional trying to get forum with GPs is very, very difficult’* (CO17).

Many participants viewed CLPs as being able to facilitate links with primary care, aiding them to *‘… get a foot in the door’* (CO13) of sites that had previously been perceived as difficult to penetrate. CLPs were perceived to carry *‘… weight, gravitas, authority and credibility with a GP practice’* (CO16) that facilitated initial contact, bypassing traditional routes. The physical location in the GP practice was thought to be of central importance for CLPs developing a working relationship with GPs. These perceptions came from the experience of actually meeting with GPs because of CLP facilitation, or from the expectation of how it may be easier to link with GPs with the CLP in place:

‘… on site and because they’re part of the mechanism of that daily practice, they’re probably going to be at meetings that I’m not going to be at, they’re probably going be hearing about patients or families or carers.’

(CO17)

CLPs were seen to have an understanding of both primary care and community organisation structure and function, and therefore could help negotiate the communication between these parties; they are *‘… in a position to champion’* (CO27). This feeling was reflected among CLPs, for example, they talked about being more sympathetic to the GP role since becoming a CLP:

‘I’ve experienced it myself as a worker, trying to get a liaison; some kind of relationship with GP surgeries is not an easy thing to do. And now working within the GP practice, you can understand some of the constraints. You know, the contract, the money, and … their surgeries are chock-a-block. So I can see why that relationship, or that link has not really grown over the years.’

(CLP2)

### Challenges to collaborative working

#### Austerity

Participants believed that austerity measures, particularly cuts to the welfare system, had brought about an increased demand for services:

‘We want to think about everybody having enough of an income that they’ll be able to provide food for them and their family. But unfortunately, the way that the welfare system is operating currently … it’s about being realistic about how the benefit system in this country is operating.’

(CO28)

However, this increase in need for services was occurring concurrently with funding cuts, whereby organisations were left with a *‘… massively reduced’* financial resource (CO18), leading to uncertainty about the future:

‘Everybody’s chasing some sort of money. Like, the women’s centre I’m talking about, they’re — they don’t know if they’re gonna be here in a year’s time. They’re chasing funding.’

(CLP4)

The CLPs saw this as potentially damaging to the services that the community organisations (with which they were attempting to build relationships with) could provide:

‘I’m seeing more and more of the time, the resources demand, the stretch on organisations in terms of the amount of people that seem to be getting referred to these organisations now. And I think potentially the quality of service of these organisations could suffer.’

(CLP6)

CLPs also noted issues with referring patients to organisations that they believed did not have sufficient capacity to support patients. This was particularly difficult to navigate because community organisations were perceived by CLPs to be hesitant to admit any lack of capacity. Confirming these concerns, smaller community organisations suggested that funding was an issue, yet also they would not turn people away:

‘We struggled to try and get funding. And so we hit a real stumbling block last year and we had somebody, something like 40 patients and I could not access funding for them … It’s just about doing that “can do” thing. It’s got nothing to do with that, you just need to believe that you’re gonna be able to make, you’re gonna have to help people. So the capacity, it’s just about the will.’

(CO3)

One issue with this approach was that organisations would tend to deal with immediate crisis cases and therefore not have resources to support those who required more long-term engagement. Community organisation representatives acknowledged that this ‘fire-fighting’ approach was not best suited to support individuals with enduring and complex health and social challenges. In recognising this, they frequently highlighted the importance of the CLP role of long-term client engagement. This approach is made viable by the CLPs’ ongoing relationship with patients.

Although austerity was viewed as a challenge to collaborative working by CLPs, community representatives unsurprisingly welcomed the extra resource, in the form of the CLP, to tackle the workload in this context:

‘It’s great that a programme like the links programme is being piloted at a time like this, which is incredibly … it’s essential, so in terms of more partnership working, getting people the right resources … especially within the current kind of political climate and framework that we’re all working within.’

(CO1)

#### The capacity of community links practitioners

Although positive in principle about roll-out of the LWP, some of the community organisation representatives voiced concern about the capacity of the CLPs given their individual case management role:

‘The CLP’s job is … a little bit difficult, shall we say? … they’re working with so many different groups of individuals with different specialist needs and, to some extent, they’re — to use a very general term — doing quite a lot of casework support. And I think that’s a big ask.’

(CO1)

Although CLPs have relative autonomy and ability to support patients at a broad level, the work is time consuming. Each of the CLPs talked about being extremely busy with supporting patients, who often have complex circumstances and are in contact in times of crisis. Although community organisation representatives recognised that this was a very intense and challenging role, they did not have particular suggestions for improving engagement with their service (for patients who they feel would benefit from it) without the case management support of the CLP.

#### Forging individual rather than organisational relationships and continuity

CLPs and most of the community organisation representatives clearly valued their collaborative relationships; however, both found it difficult to progress them to a more lasting collaboration between organisations independent of the specific individuals involved:

‘Sometimes you can have a really good relationship with an organisation, and then a worker leaves and it completely changes the dynamic. You know, you’ve built up a relationship with one person, you feel like you’ve a good sense of each other, each other’s roles, and then somebody moves on and that’s lost. And the nature of the third sector is that that’s continuous often.’

(CLP3)

Lack of continuity of staff was also a concern of community organisation representatives. Although they occasionally talked about better awareness of their service among GP practice staff, facilitated by the CLPs, they did not expect their collaborative relationship with the GP practice to continue if the CLP was no longer in post. The CLP was seen as the ‘connector’, without which access routes would be closed:

‘Our relationship didn’t really exist before and now there is a relationship. I would still say that it is mostly going through the link worker, so there’s still the need for the third party, but I don’t think that’s ever gonna change, just with the way that GP practices are structured. I think there would always need to be a link worker to be able to facilitate that relationship.’

(CO1)

The need for the CLP to facilitate links between community organisations and GP practices was not necessarily viewed as a problem by participants (both groups); as long as the CLP was in place that two-way connection could exist.

## DISCUSSION

### Summary

This study sought the views of a wide range of community organisations, providing a breadth of information on perspectives of a social prescribing programme in a primary care setting. The findings show positive views of the LWP having improved inter-sectoral working between GP practices and community organisations. However, progress towards this outcome was directly through the CLP. Community organisations’ difficulty in establishing organisational relationships with primary care, more broadly, remained apparent.

### Strengths and limitations

This study explored the LWP from the perspective of community organisations, a view not often included in evaluations of social prescribing initiatives,^24^ despite the key role they play. A range of types of organisation were included, and initial interviews with CLPs aided the formulation of the topic guide.

In identifying community organisations, CLPs were asked to name services that they had managed to create links with, as well as those where they had found making the collaboration challenging. Such collaborations were challenging because CLPs were not able to make initial contact with anyone in the organisation. For the same reason, it was difficult for the research team to recruit participants from these organisations, largely because of their nature, which was inconsistent and without permanent contactable individuals.

It is possible that views on collaboration were more positive than they might have been if community organisations were recruited without CLP input, because organisations that CLPs had been unsuccessful in making contact with were not interviewed. However, practically, recruitment was not possible any other way.

### Comparison with existing literature

Consistent with previous research, community organisation participants emphasised how difficult it had been to work collaboratively with primary care before the LWP.^28^ Such inter-sectoral working has the potential to increase social capital, one of the long-term outcomes of the LWP. Social capital is a much-contested term,^29^ but, simply, the concept is based on resources available to an individual or group, particularly from relationships involving trust, behaviour norms, social support, and information flow.^30^^,^^31^ The CLP can be seen as a ‘boundary spanner’, which has been described as a strategic brokering role that involves connecting two or more systems with partially conflicting goals or expectations.^32^^,^^33^

The full-time location of a CLP in the GP practice provided the opportunity for the CLP to become a trusted member of the GP team, where they could share information about community organisations. This co-location is also likely to facilitate easier referral from the GP, and engagement with the LWP for vulnerable patients.^17^ Previous research has hypothesised that in deprived areas this type of ‘bridging’ social capital is important for health improvement because it allows people to access resources outside of their immediate environment.^30^ The potential for increased social capital for patients in the LWP may be limited to their association with the CLP, rather than necessarily their own increased social capital. Nevertheless, being part of a ‘well-connected society’ is thought to be beneficial for individuals because they may reap some of the benefits of living in the area.^34^ There has been some progress towards inter-sectoral connections, with the CLPs developing ongoing relationships with community organisations; however, these largely rely on relationships at an individual rather than organisational level. This step is a necessary building block: sustained relationships between organisations will only be achieved through initial cultivation of social ties and individual trust.^35^ For organisational ties to be sustained, however, the relationships the CLP nurtures need to graduate to include more, and more senior, individuals from each organisation, something not yet apparent from the current study.

The CLPs in the LWP have taken on a ‘fixing’, rather than solely linking, role.^36^ The case management role of the CLPs was largely welcomed by community organisation participants because it provided a bridge between primary care and further support, and was perceived not only to facilitate access, but also ongoing engagement with further support for patients. However, given this intense and continuous involvement of CLPs in individual case management, the sustainability of the programme is unclear. CLPs are arguably filling gaps created by budget cuts elsewhere. Furthermore, the reach of the LWP may be affected because case management is time consuming, potentially limiting the number of patients that the CLP is able to engage with.^36^

The need for initiatives that provide further support to people with complex problems was thought to be timely because of the reality that people currently face, especially the strain of changes in welfare benefits and lack of employment opportunities,^37^ on top of long-term poverty and longstanding inequalities.^38^ However, funding pressures led to anxieties about community organisation capacity to support such need. These organisations are in greater demand because of the impact of austerity, therefore are more reliant on local government funding, which itself is reduced because of austerity measures.^39^ This can be thought of as a ‘perfect storm’, whereby the combination of circumstances exacerbates the situation further. To further compound the issue, evidence from England and Wales has shown that community organisations in the most deprived local authority areas have experienced a greater decline in funding.^40^ If community organisations cannot accommodate the increased level of referral from CLPs, and do not receive extra resource to manage it,^41^ the CLPs will be left to absorb the workload that GPs pass on.^42^

### Implications for research and practice

Further research is required to determine whether organisational links, independent of individual links, develop over time and whether the link worker model of social prescribing is a sustainable initiative to improve collaborative working. Given the lack of evidence about effectiveness of social prescribing interventions, high-quality evaluation of patient outcomes is required. Forthcoming articles (yet to be submitted) describing the outcome and process evaluation of the LWP, including patient views, will provide some of this evidence.

This qualitative study has shown that the implementation of the LWP has somewhat facilitated inter-sectoral working between GP practices and the community; however, challenges related to the reach and sustainability of the model remain. The role that social prescribing initiatives can play within the public health policy agenda remains unclear, particularly in the current political context of austerity. Without involving community organisations in the planning phase, to determine whether their role is realistic, and tackling funding issues that they face, the efficacy of initiatives to increase the role of community organisations in achieving public health goals is questionable.^43^

These organisations operate within the same challenges and restrictions as GP practices in areas of high socioeconomic deprivation, and are arguably a less powerful or resourced group of workers. Although social prescribing is clearly not a ‘magic bullet’,^44^ both CLPs and community organisation participants were positive in the main about the approach, and evidently there is appetite for better structures to achieve public health goals with joint working.
